# Early and Late-Phase 24 h Responses of Stored Red Blood Cells to Recipient-Mimicking Conditions

**DOI:** 10.3389/fphys.2022.907497

**Published:** 2022-06-01

**Authors:** Vassilis L. Tzounakas, Alkmini T. Anastasiadi, Dimitrios G. Karadimas, Athanassios D. Velentzas, Violetta I. Anastasopoulou, Effie G. Papageorgiou, Konstantinos Stamoulis, Issidora S. Papassideri, Anastasios G. Kriebardis, Marianna H. Antonelou

**Affiliations:** ^1^ Department of Biology, School of Science, National and Kapodistrian University of Athens (NKUA), Athens, Greece; ^2^ Laboratory of Reliability and Quality Control in Laboratory Hematology (HemQcR), Department of Biomedical Sciences, School of Health and Welfare Sciences, University of West Attica (UniWA), Egaleo, Greece; ^3^ Hellenic National Blood Transfusion Centre, Athens, Greece

**Keywords:** *in vitro* model, transfusion medicine, red blood cells, hemolysis, RBC morphology

## Abstract

The 24-hour (24 h) post-transfusion survival of donor red blood cells (RBCs) is an important marker of transfusion efficacy. Nonetheless, within that period, donated RBCs may encounter challenges able to evoke rapid stress-responses. The aim of the present study was to assess the effect of exposure to plasma and body temperature upon stored RBCs under recipient-mimicking conditions *in vitro* from the first hours “post-transfusion” up to 24 h. For this purpose, packed RBCs from seven leukoreduced CPD/SAGM units were reconstituted with plasma of twenty-seven healthy individuals and incubated for 24 h at 37^o^C. Three units were additionally used to examine stress-responses in 3-hour intervals post mixing with plasma (*n* = 5) until 24 h. All experiments were performed in shortly-, medium-, and long-stored RBCs. Hemolysis, redox, morphology, membrane protein binding and vesiculation parameters were assessed. Even though spontaneous hemolysis was minimal post-reconstitution, it presented a time-dependent increase. A similar time-course profile was evident for the concentration of procoagulant extracellular vesicles and the osmotic fragility (shortly-stored RBCs). On the contrary, mechanical fragility and reactive oxygen species accumulation were characterized by increases in medium-stored RBCs, evident even from the first hours in the recipient-mimicking environment. Finally, exposure to plasma resulted in rapid improvement of morphology, especially in medium-stored RBCs. Overall, some RBC properties vary significantly during the first 24 h post-mixing, at levels different from both the storage ones and the standard end-of-24 h. Such findings may be useful for understanding the performance of RBCs and their possible clinical effects −especially on susceptible recipients− during the first hours post-transfusion.

## Introduction

Transfusion of red blood cells (RBCs) is one of the most common medical procedures worldwide, therefore its optimization is always in the spotlight. Stored RBCs undergo a series of alterations, collectively known as storage lesion, which affect their morphology and metabolism and may impact their post-transfusion efficacy (Yoshida et al., 2019). A part of the storage lesion can be reversed once in the circulation of the recipient, as in the case of ATP depletion (Barshtein et al., 2018) or mild morphological modifications, but some phenotypes, like membrane loss through vesiculation and acquisition of spherocytic morphology, are considered irreversible (Prudent et al., 2015). Moreover, features like the osmotic or mechanical fragility hint the presence of sublethal injuries on the RBC membrane; namely, storage related injuries that render cells more prone to removal/lysis once transfused (Antonelou and Seghatchian, 2016).

The two gold standards of transfusion are end-of-storage in-bag hemolysis and 24 h post-transfusion recovery. Nonetheless, recovery is the final phenotype, thus this parameter does not give information regarding the underlying reason for the removal of RBCs or the exact time within the 24 h that this event occurred. In this context, a variety of factors have been associated with the outcome of transfusion therapy. For example, deformability of transfused RBCs presents a highly significant positive correlation with the hemoglobin (Hb) increment in thalassemic patients (Barshtein et al., 2017). Additionally, the levels of some metabolites have been found related to the post-transfusion survival of RBCs *in vivo*: hypoxanthine has been linked to reduced recovery (Nemkov et al., 2018), while octenoyl-carnitine to increased recovery (Francis et al., 2020).

To study the physiology of transfused RBCs, *in vitro* conditions that mimic some aspects of the recipient’s environment have been used. Stored RBCs have been exposed to body temperature (Roch et al., 2019) and healthy, diseased or pro-inflammatory plasma (Mittag et al., 2015; Tzounakas et al., 2016; Anastasiadi et al., 2021; Längst et al., 2021) to examine the impact of temperature transition and plasma components upon these cells. There are also *in vitro* models that aim to unravel immunologic derangements, by exposing stored RBCs to human T and B cells (Long et al., 2014). While they cannot completely simulate the recipient’s circulation, these models have proven to be useful and reliable, since results obtained by their usage are consistent with respective analyses in animal models or clinical trials, as exemplified in the case of glucose-6-phosphate deficient and beta-thalassemia minor donors (Tzounakas et al., 2016; Francis et al., 2020; Anastasiadi et al., 2021).

Having all the above in mind, the aim of the present study was to examine the physiology of shortly- medium- and long-stored RBCs when in contact with plasma at body temperature, already from the first hours of *in vitro* “transfusion” until the commonly studied 24 h, to get insight into 1) the timing of a series of RBC alterations “post-transfusion” and 2) the degree of changes in RBC physiology after transition from the cold to a recipient simulating environment.

## Materials and Methods

### Biological Samples and Blood Unit Preparation

Seven leukoreduced RBC units containing citrate-phosphate-dextrose (CPD)/saline-adenine-glucose-mannitol (SAGM) were prepared from healthy individuals and were stored for 42 days at 4^o^C. Sampling was performed under aseptic conditions in early (day 2; shortly-stored RBCs), middle (day 21; medium-stored RBCs) and late (day 42; long-stored RBCs) storage. Fresh blood was drawn by twenty-seven healthy donors into citrate vacutainer tubes. The study was approved by the Ethics Committee of the Department of Biology, School of Science, NKUA. Investigations were carried out upon donor consent, in accordance with the principles of the Declaration of Helsinki.

### Recipient Environment Simulation

To assess the effect of plasma and body temperature upon stored RBCs an *in vitro* model was used (Anastasiadi et al., 2021). Shortly-, medium- and long-stored RBCs were reconstituted in healthy plasma from twenty-seven potential control recipients which was previously mixed with each unit’s supernatant to finally reach a ratio analogous to the administration of two blood units (32–34% hematocrit). The reconstituted samples were incubated for 24 h at 37^o^C and in 5% CO_2_-air and were under constant gentle agitation to avoid settling. RBCs from three of the units, mixed with plasma from five subjects, were additionally examined in 3-hour intervals post reconstitution, up to 24 h, thus an aliquot was prepared for every time-point under examination. Results from the 24 h time point of reconstitution (*n* = 27) were integrated with those of 3-hour intervals (*n* = 5) in all figures to simplify the structure and the presentation of the manuscript. Assays of hemolysis, ROS accumulation, potassium release, procoagulant EV activity, RBC morphology and protein detection were performed. All assays were performed in 3-hour intervals unless otherwise stated. All measurements were also made in stored RBCs of each unit of origin at blood bank conditions.

### Hemolysis Parameters

In-bag and spontaneous hemolysis (levels of extracellular Hb) were calculated by spectrophotometry, using Harboe’s method (Harboe, 1959) followed by Allen’s correction. To assess the susceptibility to osmotic stress, the samples were exposed to ascending concentrations of NaCl (0–0.9%) and thereafter the mean corpuscular fragility (MCF) index was calculated (i.e., %NaCl at 50% hemolysis). 1 h rocking (18 rpm; rocking angle ±17^o^) of samples with stainless steel beads was used to implement a mechanical stimulus. To consider only the mechanically induced hemolysis, non-rocked aliquots served as baseline-hemolysis controls under the same conditions. After two sequential centrifugations (2750xg/15 min and 20,800xg/20 min) extracellular Hb levels were measured (Harboe’s method). The levels of total Hb concentration were also measured and the mechanical fragility index (MFI) was calculated as follows: MFI(%) = [(Hb_(rocked)_—Hb_(non-rocked)_)/(Hb_(20%)_ –Hb_(non-rocked)_)] x 100 (Raval et al., 2010). Oxidative hemolysis (performed in 3, 6, 12 and 24 h) was evaluated upon treatment of RBCs with 17 mm phenylhydrazine (PHZ) for 1 h at 37^o^C (Tzounakas et al., 2022), before centrifugation and measurement of the Hb released in the supernatant (Harboe’s method).

### ROS Accumulation

Intracellular accumulation of ROS was fluorometrically measured using the membrane permeable and redox-sensitive probe 5-(and-6)-chloromethyl-2′,7′-dichloro-dihydro-fluoresceindiacetate, acetyl ester (CM-H_2_DCFDA) in intact RBCs. Intracellular esterases cleave this molecule, making it unable to exit, and upon its oxidation by ROS it yields the fluorescent dichlorofluorescein (DCF). Briefly, small aliquots of RBCs were loaded with 10 μmol/L CM-H_2_DCFDA (30min/room temperature). A wash was then performed, followed by a short recovery time (12min). The cells were finally lysed to measure the fluorescence levels. Apart from the intrinsic ROS, the oxidative burden of RBCs was also evaluated after exogenous stimulation with tert-butyl hydroperoxide (tBHP; 100 μM), diamide (2 mm) and PHZ (100 μM; performed in 3, 6, 12 and 24 h). This was performed in order to study the effects of additional oxidative stress upon ROS accumulation, especially since some potential transfusion recipients might be characterized by increased oxidative burden. Diamide and phenylhydrazine mainly target glutathione and Hb, respectively, while tBHP is a more general oxidative reagent. Quantification of ROS levels was achieved after normalization to protein concentration (Tzounakas et al., 2022).

### Potassium Leakage and Extracellular Vesicles’ Procoagulant Activity

Biochemical analysis of potassium (K^+^) was performed in the supernatant of samples reconstituted for 3, 6, 9, 12, 18, and 24 h using the AVL Series Electrolyte Analyzer 9,180. Extracellular vesicles’ (EV) procoagulant activity was measured for the same time points using a functional ELISA assay kit (Zymuphen MP-activity, Hyphen BioMed, Neuvillesur-Oise, France), as per manufacturer’s specifications. This assay is based on the conversion of prothrombin to thrombin in the presence of PS^+^ EVs in the supernatant of the samples and the addition of coagulation factors, Ca^2+^ and prothrombin. Using a chromogenic thrombin substrate (absorbance at 405 nm) and a standard curve, the concentration of procoagulant EVs can be calculated in nM of PS.

### Scanning Electron Microscopy

Morphological evaluation of stored (medium-long storage) and reconstituted (6, 12, and 24 h) RBCs was based on scanning electron microscopy. Purified RBCs were firstly fixed with 2% glutaraldehyde and post-fixed with 1% osmium tetroxide in 0.1 mol/L sodium cacodylate buffer, pH 7.4. To dehydrate the samples before coating with gold-palladium, they were exposed to ascending ethanol concentration solutions. Electron micrographs (x1,000) were taken at random fields and cells with spherocytic modifications (spherocytes, spheroechinocytes, spherostomatocytes) or degenerative shapes were categorized as “irreversible”. At least 2,000 cells were blindly evaluated for each sample.

### Membrane Isolation and Immunoblotting Analysis

RBC membranes were isolated by hypotonic lysis (Kriebardis et al., 2007) of old reconstituted (for 3, 6, 12, and 24 h) RBCs in 5 mmol/L sodium phosphate buffer, as previously extensively described. Membrane aliquots (30 μg) were immune-probed for a variety of proteins (known to be potentially recruited to the RBC membrane) by using horseradish peroxidase-conjugated secondary antibodies and enhanced chemiluminescence development. Primary antibodies against calpain, soluble clusterin, HSP70 (Santa Cruz Biotechnology, Santa Cruz, CA, United States), peroxiredoxin-2 (Acris, Luzern, Switzerland), human Hb (Europa Bioproducts, Wicken, UK), human IgGs (Sigma Aldrich, St. Louis, MO, United States) and caspase-3 (Cell Signaling Technology, Danvers, MA, United States) were used. Antibody against 4.1 R was kindly provided by Prof. J. Delaunay (Laboratoire d’ Hématologie, d’Immunologie et de Cytogénétique, Hopital de Bicetre, Le Kremlin-Bicetre, France). The bands were quantified by scanning densitometry (Gel Analyzer v.1.0, Athens, Greece).

### Statistical Analysis

All experiments were performed in duplicate. Statistical analysis was performed by using the statistical package SPSS Version 22.0 (IBM Hellas, Athens, Greece, administered by NKUA). Repeated measures ANOVA with Bonferroni-like adjustment for multiple comparisons was used for the evaluation of time-course and between groups differences. Significance was accepted at *p* < 0.05.

## Results

**FIGURE 1:**
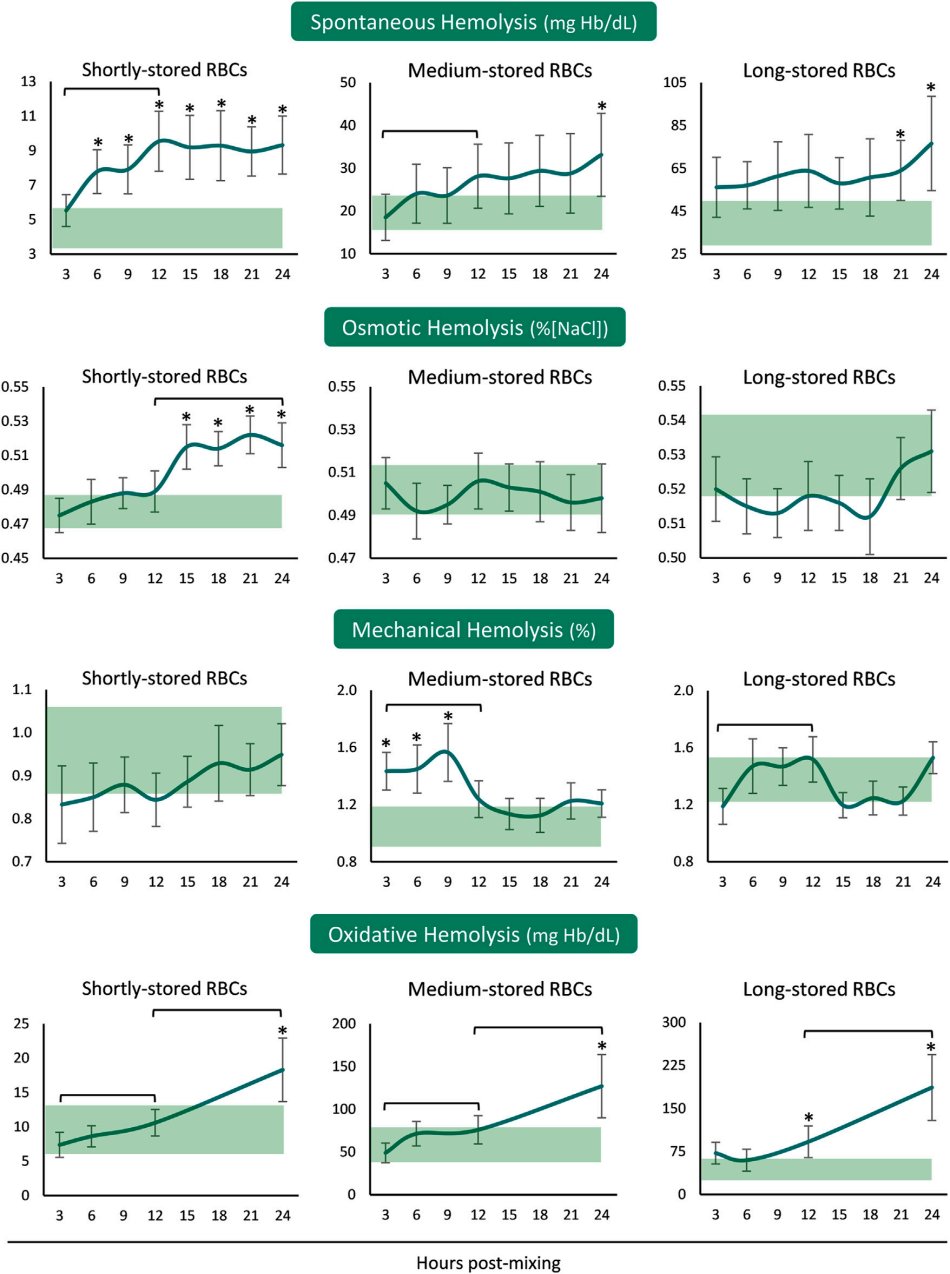
Hemolysis parameters of reconstituted stored red blood cells in freshly drawn plasma. Baseline levels of stored RBCs in the cold (4°C) are shown by transparent horizontal bands (band thickness is representative of the standard deviation [SD]). (*) *p* < 0.050 reconstituted RBCs vs. baseline RBCs in the cold; brackets show differences with *p* < 0.050 between 3, 12, and 24 h post reconstitution.

In the presence of plasma and body temperature, shortly- and medium-stored RBCs presented an increase in hemolysis within the first 12 hours, but not later. Released hemoglobin concentration was greater than the in-bag one in shortly-stored RBCs at all time-points after reconstitution but only when nearing 24 h in the medium- and long-stored RBCs (Figure 1). The time-course patterns of mechanical and osmotic fragilities varied between the RBCs of different storage age post reconstitution. Interestingly, the shortly-stored cells presented increased osmotically induced hemolysis from 12 h onwards, at levels exceeding the storage ones. On the other hand, medium-stored RBCs were characterized by higher mechanical fragility compared to the storage levels, already from the first hours and until the 12 h interval in the recipient simulating environment. Oxidative (PHZ) hemolysis was consecutively increasing post reconstitution towards values higher than those of the blood bag in 24 h, regardless of storage age (Figure 1).

**FIGURE 2:**
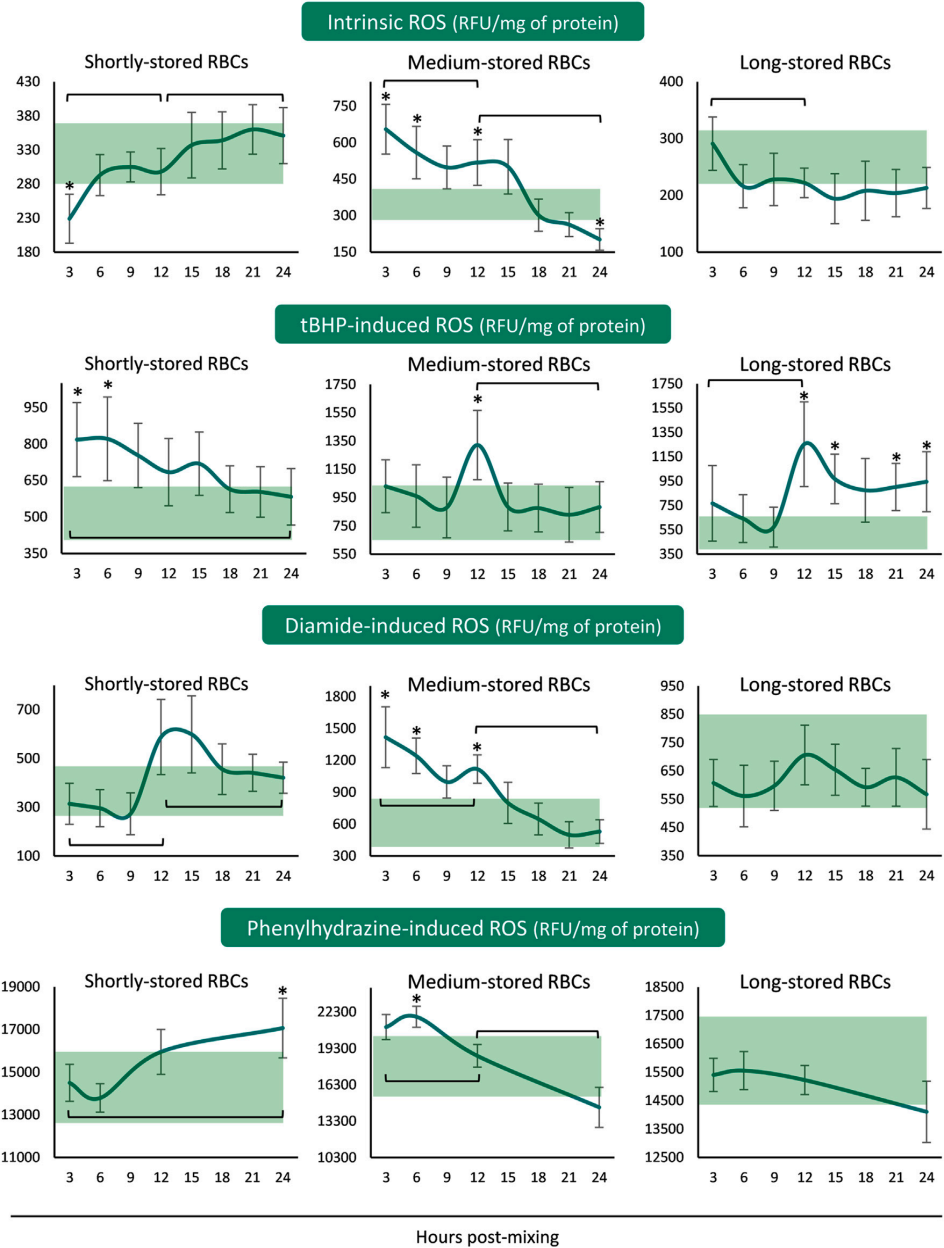
Reactive oxygen species (ROS) of reconstituted stored red blood cells in freshly drawn plasma with or without external stimuli. Baseline levels of stored RBCs in the cold (4°C) are shown by transparent horizontal bands (band thickness is representative of the standard deviation [SD]). (*) *p* < 0.050 reconstituted RBCs vs. baseline RBCs in the cold; brackets show differences with *p* < 0.050 between 3, 12, and 24 h post reconstitution.

The ROS accumulation profiles differed for every storage period tested. In shortly-stored RBCs intrinsic and diamide- or PHZ-induced ROS presented a rising trend from the beginning until the end of the 24 h incubation period, with the levels not really deviating from the corresponding storage ones (Figure 2). The exact opposite pattern was revealed when triggered with tBHP: intracellular ROS accumulation was higher than inside the unit for the first 6 h and then reached similar levels. Medium-stored RBCs presented the highest ROS values, both time-course and compared to the unit, already from the first 3–6 h (intrinsic, diamide- and PHZ-induced) or at 12 h (tBHP-induced) post-reconstitution. Lastly, in long-stored RBCs only the tBHP-induced ROS increased at higher than storage levels around the half of the incubation period, as opposed to the other three conditions that exhibited variations within the storage levels (Figure 2).

**FIGURE 3:**
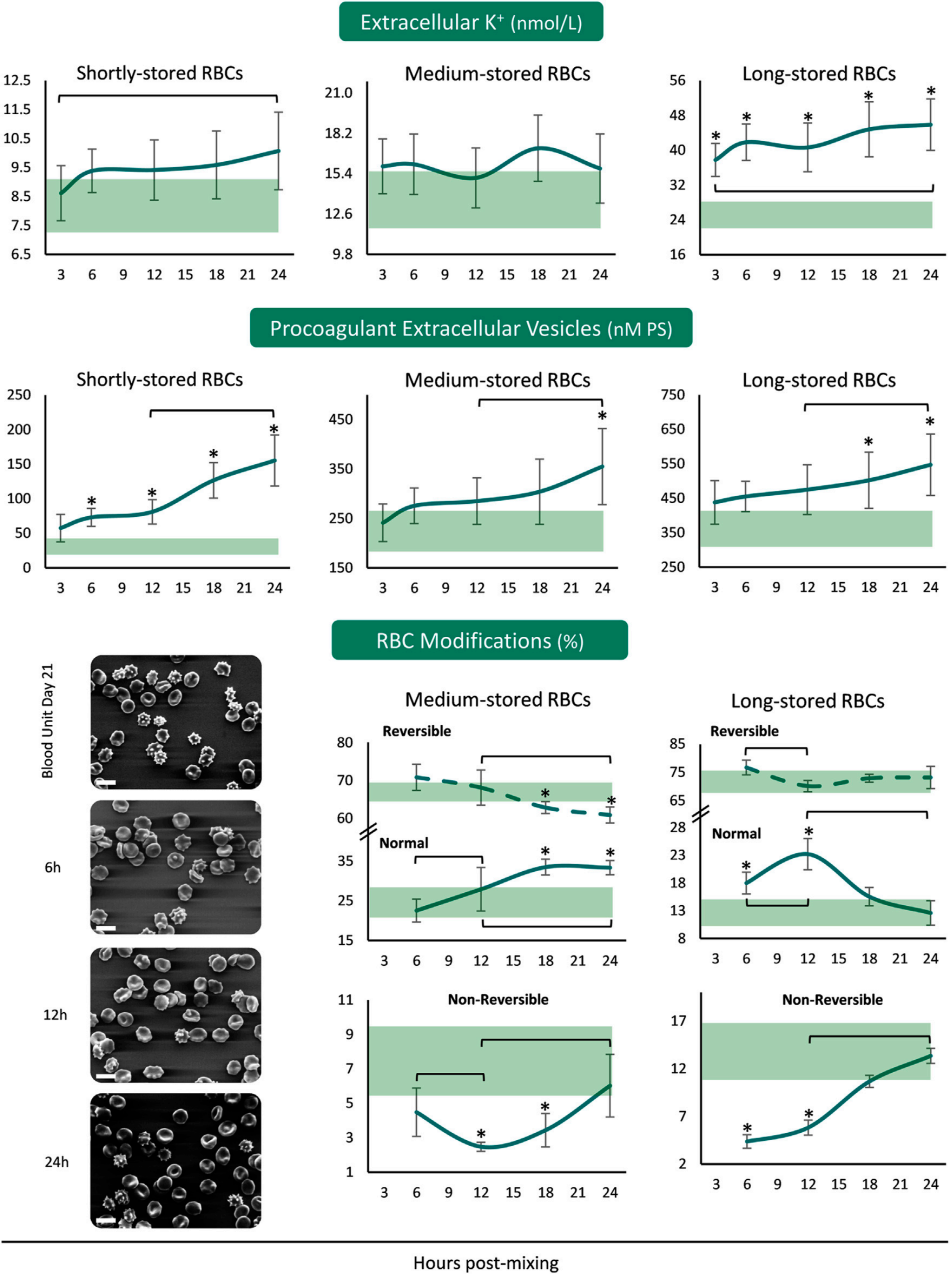
Potassium leakage, extracellular vesicles’ procoagulant activity and RBC morphology of reconstituted stored red blood cells in freshly drawn plasma. Baseline levels of stored RBCs in the cold (4°C) are shown by transparent horizontal bands (band thickness is representative of the standard deviation [SD]). (*) *p* < 0.050 reconstituted RBCs vs. baseline RBCs in the cold; brackets show differences with *p* < 0.050 between 3 h (or 6 h in the case of RBC modifications), 12 and 24 h post reconstitution. Representative micrographs from scanning electron microscopy are shown for day 21 samples (x 1,000); white scale bars: 10 μm.

The concentration of extracellular K^+^ was increasing from the beginning until the end of the 24 h period in the plasma of both shortly- and long-stored reconstituted samples, with higher than storage levels in the reconstitutions of long-stored RBCs (Figure 3). Concerning the release of procoagulant EVs from the reconstituted RBCs, there were increasing patterns in all the samples from 12 to 24 h. There were more PS^+^ EVs in the reconstituted samples of shortly-stored RBCs compared to the units of origin, already from 6 h onwards, but later on in the reconstitutions of medium- and long-stored RBCs. Transition of medium-stored RBCs to plasma environment at body temperature resulted in a gradual increase in normal discocytes during the 24 h period at the expense of reversible RBC modifications. In addition, the percentage of discocytes was higher than the one in-bag from 12 h post-reconstitution onwards, while irreversible shape modifications were steadily lower compared to the storage levels. In the samples of long-stored RBCs lower irreversible modifications and more discocytes (compared to the storage levels) were detected the first 12 h post reconstitution (Figure 3).

**FIGURE 4:**
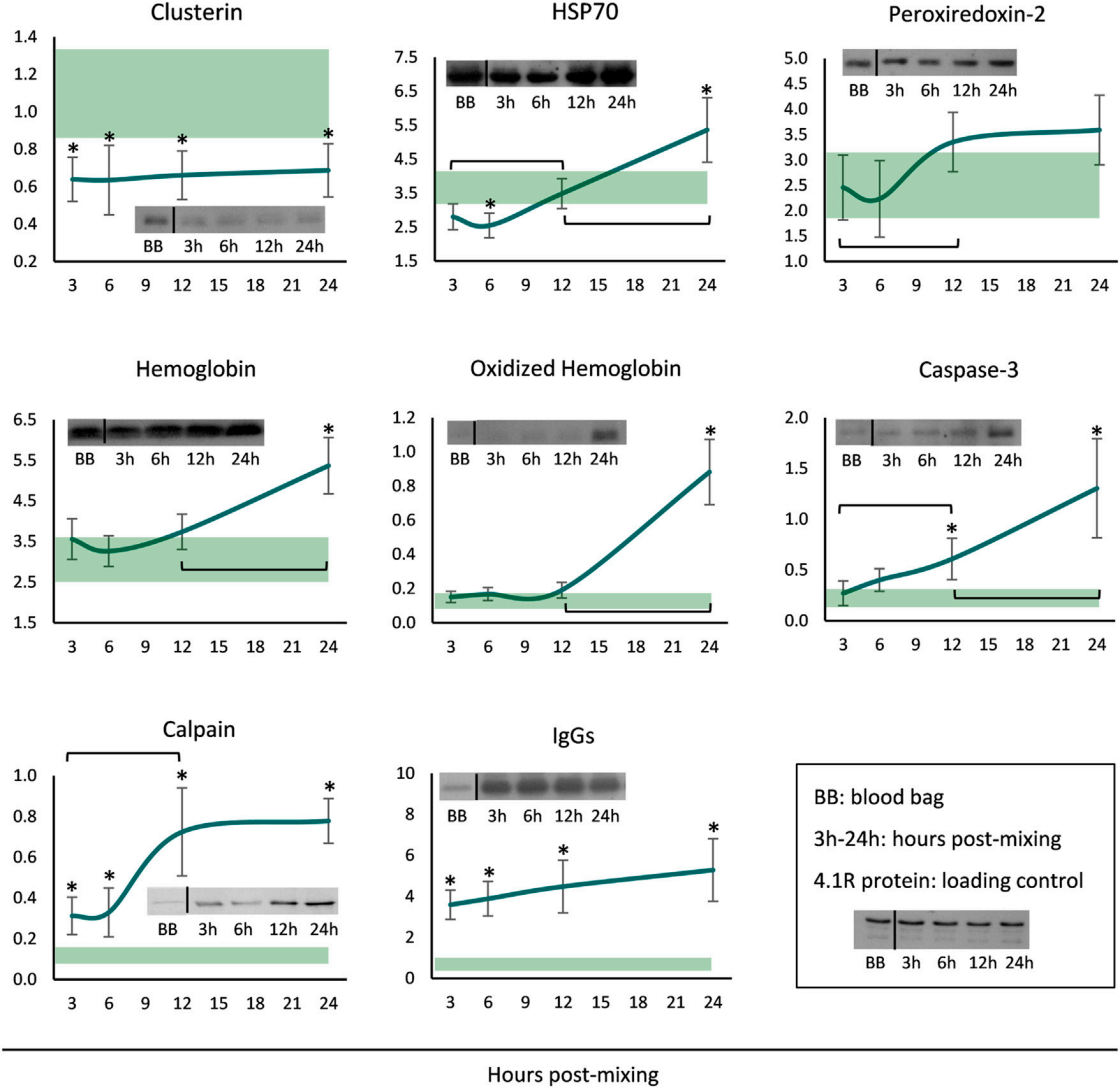
Protein binding profile (arbitrary units after normalization to 4.1 R protein levels) of reconstituted long-stored red blood cells in freshly drawn plasma. Baseline levels of stored RBCs in the cold (4°C) are shown by transparent horizontal bands (band thickness is representative of the standard deviation [SD]). (*) *p* < 0.050 reconstituted RBCs vs. baseline RBCs in the cold; brackets show differences with *p* < 0.050 between 3, 12 and 24 h post reconstitution; inserts: indicative western blots of the selected proteins.

Finally, membranes of long-stored reconstituted RBCs were probed for a variety of potentially membrane-binding proteins. The levels of clusterin were steady throughout the incubation period, but always lower when compared to the storage levels (Figure 4). Starting by very low levels, HSP70 binding gradually increased and maximized at 24 h. Increased peroxiredoxin-2 (prdx2) levels were also evident around the middle of the incubation period, but the levels were similar to those in the cold. Membrane-associated hemoglobin, its oligomers as well as caspase-3, commenced at storage levels but significantly increased from middle incubation onwards. Finally, the membrane binding of calpain and IgG immunoglobulins was always higher in the reconstituted RBCs compared to storage, and additionally, in the first case, a time-course increase was evident from 3 to 12 h (Figure 4).

## Discussion

Since the 24 h RBC recovery is one of the gold quality standards of transfusion efficacy, this specific time point has been thoroughly studied in clinical trials as well as in numerous *in vivo* and *in vitro* models (de Wolski et al., 2016; Francis et al., 2020; Anastasiadi et al., 2021). Hereby, by using an *in vitro* simulation of transfusion we provide evidence regarding the time-course of physiological changes that occur in stored RBCs, as they reach the first 24 h post mixing with plasma at body temperature. Our results showed that 1) some parameters of medium-stored RBCs, such as mechanical fragility and ROS accumulation, present an early response to the new conditions by reaching their highest values, exceeding the storage levels, the very first hours post mixing, 2) transition to recipient-mimicking conditions mainly preserves RBC integrity by favoring their morphology while keeping spontaneous intravascular hemolysis to minimal, and 3) the binding of several stress-related proteins to the membrane of long-stored RBCs is gradually elevated 24 h post-mixing with recipient plasma, and, in some cases, already half way there.

When incubated with plasma at body temperature, stored RBCs were characterized by extremely low loss of Hb in comparison to the respective *ex vivo* storage conditions. Even in shortly-stored reconstituted RBCs, that were found more prone to spontaneous hemolysis compared to baseline, the new production of free Hb was extremely low, not capable of leading to post-transfusion complications to the recipient. Considering the *in vitro* nature of these experiments, which may also lead to exacerbated hemolysis during prolonged incubation (Längst et al., 2021) due to continuous absence of pro-survival factors, such findings are in line with studies reporting that transfused RBCs are cleared from the circulation through extravascular rather than intravascular hemolysis (Hod et al., 2011). At the same time, the low levels of hemolysis post-reconstitution with plasma, when compared to the higher susceptibility to spontaneous lysis observed by *in vitro* models examining temperature-related RBC modifications in its absence (Burger et al., 2013), highlights the protective effect of plasma components on transfused RBCs (Butler et al., 1992). The osmotic hemolysis of shortly-stored RBCs followed a distinct pattern of gradual increase during the 24 h incubation period towards levels seemingly equal to those of long-stored RBCs. It is plausible to suppose that the loss of K^+^ that characterizes long-stored RBCs (Vraets et al., 2011), even under the currently presented reconstitution conditions, causes a decrease in cell volume that enables these cells to effectively cope with water influx, counteracting the simultaneously observed membrane loss. Whatever the mechanism is, two separate issues arise. Firstly, long-stored RBCs release a high amount of potassium cations to the extracellular environment right after their contact with recipient’s plasma or the transition to 37^o^C (Burger et al., 2013), which can be harmful to specific patient backgrounds (e.g., neonates) post-transfusion. In fact, it has been reported that clinical hyperkalemia and cardiac arrests can occur after transfusion of long/many stored RBC units (Vraets et al., 2011). Secondly, it seems that RBCs stored for only 2 days, namely the closest to the *in vivo* condition available, present an increased rate of lesions post-mixing, as in the case of osmotic fragility or extracellular vesicle release. These features should be also taken into consideration for transfusions in specific recipient groups, like those with renal deficiencies, that affect the osmotic balance (Dhondup and Qian, 2017), or patients with coagulation issues (Kvolik et al., 2010), respectively. It should be noted that these RBCs have experienced severe environmental changes in only 48 h, such as transition from body temperature to 4^o^C and *vice versa* as well as filter leukoreduction. Whether this procedure stresses them leading to augmented RBC vesiculation rate, as reported for specific donor contexts (Hess et al., 2009), remains to be determined.

The study of medium-stored RBCs upon reconstitution provided some of the most interesting results of this study. RBCs stored for 21 days presented an impressive and rapid improvement of their morphology as evidenced by scanning electron microscopy evaluation. The parallel increase in discocytic forms at the expense of reversible shape modifications, along with the early decrease of irreversible RBCs, point towards the favorable effect of plasma albumin, a component related to the preservation of morphological and hemorheological features (Reinhart et al., 2015). The slightly decreased levels of irreversible modifications might be attributed to their rupture, as evident by the low levels of Hb release. In addition, some RBCs categorized as irreversible might instead be type III echinocytes (Antonelou et al., 2012), that are on the verge of becoming irreversible or not. Notably, the first 12 h when the percentage of reconstituted discocytes is in the range of stored samples, the susceptibility to mechanical stress is significantly elevated. This finding is rather anticipated due to the irreversible membrane and cytoskeletal injuries that affect the structural stability, and firstly appear in medium-stored RBCs (Kozlova et al., 2021). It is tempting to hypothesize that, when transfused, the irreversibly transformed medium-stored RBCs are rapidly cleared in the spleen clefts as a result of their increased mechanical instability, currently observed in the first hours post exposure to recipient conditions. Although long-stored RBCs start with the same post-mixing pattern, they are not able to keep up until 24 h *in vitro*, probably due to the observed 1) extensive loss of membrane through vesiculation and 2) insults to their membrane, evidenced by the time-dependent recruitment of cytosolic components which belong to the “repair or destroy box” of proteins (Goodman et al., 2007; D'Alessandro et al., 2010), along with proteases. Interestingly, this progressive recruitment of those proteins during the first 24 h post-mixing with recipient plasma, hereby shown for the first time, resembles the one that occurs in stored RBCs within their 42-day lifespan in-bag. Such a shift, evident from the first 12 h in some cases, is indicative of the need to keep up with proteostatic and redox imbalances as well as of an array of insults to the RBC integrity. On the one hand the HSP70 system, that accounts for ∼1/3 of the RBCs’ chaperome and is responsible for prevention of protein aggregation, protein disaggregation and protein refolding (Mathangasinghe et al., 2021), translocates to protect membrane proteins. Accordingly, the antioxidant peroxiredoxin-2 when bound to the membrane seems to ameliorate the detrimental effects of oxidized Hb (Cha et al., 2000). On the other hand, the recruitment of caspase-3 is well known for promoting the cleavage of band 3 cytoplasmic domain in both stored (Rinalducci et al., 2012) and aged RBCs (Mandal et al., 2003) as a consequence of oxidative stress. In the same context, calpain presence to membrane fractions may lead to degradation of ankyrin and other cytoskeletal components (Mortensen and Novak, 1992) resulting in impaired deformability as previously shown in both knockout (Wieschhaus et al., 2012) and sickle cell disease mouse models (De Franceschi et al., 2013). Taken together, these observations point towards the accumulation of stressful factors to the membrane already from the first 12 h post-mixing. Whether this recruitment is homeostatic, or it mainly acts as a stress response without beneficial outcome, remains to be elucidated.

The pattern of intracellular ROS production was also interesting, showing a rapid accumulation of ROS in medium-stored RBCs the first hours post mixing. Middle storage is a key time point for RBCs regarding the development of metabolic and oxidative lesions (Reisz et al., 2016; Tzounakas et al., 2021; Tzounakas et al., 2022). At this time period a metabolic shift is observed, and the cells present high (in several cases the highest) ROS production accompanied by extensive oxidative defects to membrane lipids and proteins. These changes seem to render the cells more susceptible to alterations in their intracellular oxidative burden when exposed to a “closer to normal” environment. *In vitro* studies on the transition of stored RBCs from cold to body temperature (without the addition of plasma) revealed a decrease in NADPH levels as well as dimerization of Prdx2 from middle storage onwards (Roch et al., 2019). The only exogenous oxidative stimuli that induced a distinct ROS generation profile post-reconstitution (namely rapid accumulation in both shortly- and medium-stored RBCs) was tBHP, a general reagent that hits numerous cellular targets. Such findings might be of great importance for patients under redox-reactive medication or with distinctive redox profile (e.g., thalassemia major; (Fibach and Dana, 2019)) in need of transfusion therapy. In the first case, there is a wide spectrum of clinically evaluated drugs that inhibit redox signaling, such as glutathione inhibitors (Raza et al., 2009; Kirkpatrick and Powis, 2017) used to treat cancer or myelodysplastic syndromes in potential transfusion recipient patients. In the second case, the transfusion-dependent thalassemia major patients are characterized by excess of the redox-active iron in their circulation (Hershko, 2010). Regarding oxidative hemolysis, the shortly- and medium-stored reconstituted RBCs exhibited maximum values in the commonly studied end point of 24h, though, their older counterparts had above storage levels from 12 h onwards. While the antioxidant effect of plasma is well-known (Yeum et al., 2004), the fact that the incubation took place *in vitro*, namely, in the absence of clearance mechanisms, endothelium and normal blood flow, may lead to the accumulation of lesions on the RBC membrane. Interestingly, the time-course pattern of the oxidatively-induced hemolysis was similar to that of the membrane-bound Hb and Hb species in long-stored RBCs. Owning to the pro-oxidant activity of the hemin and Heinz bodies (Chiu and Lubin, 1989), the increased affinity of oxidized/denatured Hb for membrane docking sites results in lipid peroxidation and protein carbonylation (Bose et al., 2022). Another fine example of membrane attachment was that of plasma-recruited IgGs to reconstituted longer-stored RBCs, a finding suggestive of their rapid clearance post-transfusion due to RBC opsonization. In the same context, prolonged storage leads to increased phosphatidylserine exposure, another “non-self” signal of RBCs, after transition to recipient environment conditions *in vitro* (Burger et al., 2013; Mittag et al., 2015). Paradoxically, clusterin recruitment was not observed in the membrane of long-stored reconstituted RBCs, since its bound levels were significantly lower compared to those in the cold. Even though this finding needs further evaluation, it clearly suggests that the clusterin load of stored RBC membrane represents more than a passive recruitment from the plasma circulating stores.

Stored RBCs, whilst having accumulated an array of storage lesions, must cope with additional stresses when transfused, which may further burden their physiology. Although the new environment is recipient-dependent with respect to the clearance mechanisms or the inflammatory and redox aspects, the transfused RBCs will encounter, in all cases, baseline temperature-, plasma- and mechanical stress-related challenges. The currently presented transfusion model can shed some light regarding the occurrence and time course of RBC alterations post exposure to plasma and body temperature. We hereby report for the first time some rapid cellular responses to those newly encountered challenges that might prove to be clinically relevant in terms of post-transfusion recovery and adverse reactions to the recipient. The closed-system nature of this *in vitro* model might exacerbate some of the observed alterations, especially due to the absence of removal mechanisms, neighboring cells, and survival signals. Despite this limitation, we believe that its application to different recipient settings and post-mixing times can provide useful information regarding stored RBC performance after transfusion.

## Data Availability

The raw data supporting the conclusions of this article will be made available by the authors, without undue reservation.
